# Effects of Wuxi CDC WeChat official account article features on user engagement in health promotion

**DOI:** 10.1186/s12889-024-18277-4

**Published:** 2024-03-11

**Authors:** Xinyi Yin, Junxia Pan, Fanfan Xu

**Affiliations:** Department of Health Promotion, The Affiliated Wuxi Center for Disease Control and Prevention of Nanjing Medical University, Wuxi Center for Disease Control and Prevention, 499 Jincheng Road, 214023 Wuxi, China

**Keywords:** Health education, Information dissemination, Communication effectiveness, WeChat official account, Social media health promotion

## Abstract

**Objective:**

To identify the characteristics of subscribers to assess users’ needs and analyze the features of articles published on Wuxi CDC WeChat official account (WOA) to evaluate the effectiveness of health education dissemination and guide future communication strategies.

**Methods:**

Collect data from the WeChat official account (WOA) of the Wuxi Center for Disease Control and Prevention (CDC) to identify factors affecting the effectiveness of health education dissemination as measured by shares and 100% reading completion rate between January 1, 2022, and December 31, 2022. Multivariate logistic regression analysis was utilized to identify influencing features of articles associated with health education dissemination.

**Results:**

By the end of 2022, our account had accumulated 891,170 subscribers, of which, 523,576 were females (58.75%), 349,856 were males (39.3%), mainly located in third-tier cities (82.59%). Age distribution peaked in the 26–35 and 36–45 age groups (43.63% and 30.6%, respectively). A total of 170 articles were included in the analysis. Multivariate logistic regression analysis revealed that articles with a lower word count (OR = 0.999, 95% CI = 0.998 ~ 1), lower picture count (OR = 0.892, 95% CI = 0.828 ~ 0.962), dominated headlines (OR = 2.454, 95% CI = 1.234 ~ 4.879) and thematically focused on Nutrition and food-borne diseases (OR = 5.728, 95% CI = 1.778 ~ 18.458) demonstrated higher engagement, as measured by shares and 100% completion rates.

**Conclusions:**

Our findings suggest that future content should prioritize conciseness, optimize images, and align with subscriber interests, particularly in nutrition and food hygiene. Additionally, maintaining informative yet engaging content formats remains crucial for maximizing reach and impact.

**Supplementary Information:**

The online version contains supplementary material available at 10.1186/s12889-024-18277-4.

## Introduction

### Background of WeChat official account (WOA)

With the unprecedented growth of emerging media, such as Internet platforms, the escalating demand for health information has necessitated novel approaches in health education, presenting new opportunities [1, 2]. WeChat, a multifaceted platform offering services ranging from instant messaging, free phone calls, interest or private groups, to information sharing via moments, mobile payments, and red envelope exchanges [3], has witnessed rapid popularity, emerging as the foremost information-sharing platform in China. As of the first quarter of 2020, it boasts over 1.2 billion monthly active users worldwide [4, 5]. The extensive public engagement on WeChat establishes a robust foundation for the effective online distribution and diffusion of information by health promotion agencies [6]. The WeChat public platform, a functional module enabling operators to furnish information and services through public accounts to WeChat users [7–9], has gained widespread adoption in medical and health institutions in recent years due to its online social networking and information dissemination functionalities [10, 11]. WeChat official account (WOA) is an application account supplied by administrators on the WeChat public platform, facilitating communication and interaction with specific groups through text, pictures, voice, and video. Public members can follow WOAs of interest to receive pertinent information or messages. The WOA of the CDC is considered as an appropriate and professional platform for informing, educating, and empowering the public regarding health issues [12, 13]. In line with this, the Wuxi CDC established its WeChat official account in 2015, with the objective of disseminating disease prevention knowledge and offering public health services to the community.

### Change of WeChat official account (WOA) content

During the outbreak of the COVID-19 pandemic, there has been an increased attention to health and the frequency of public social media use because of pandemic-related quarantine and movement limitation measures [14]. WOAs provided necessary medical support for the public, reduced social panic, promoted social isolation, enhanced the self-protection ability of the public, and promoted epidemiological screening, thereby playing an important role in preventing and controlling the spread of COVID-19 [15]. Prior to 2022, the Wuxi CDC WOA primarily disseminated COVID-19 content, such as guidelines and disease updates.

In response to China’s evolving COVID-19 policy in 2022, the content disseminated by the Wuxi CDC WOA shifted from predominantly relaying official pandemic updates to a broader mandate encompassing comprehensive disease prevention, health education, and health promotion. Drawing upon the Health Belief Model and Diffusion of Innovations theory, we aim to investigate two key research questions: (1) What are the demographic and behavioral characteristics of the Wuxi CDC subscriber base? (2) Which article features, such as topic, length, and visual elements, influence user engagement with non-COVID-19 health education content? By analyzing articles published in 2022 that fall outside the purview of pandemic-related information, we aim to generate valuable insights that can inform the future direction of the Wuxi CDC WOA’s health education operations in the post-pandemic landscape.

## Methods

### Data collection and logging

Data were collected anonymously from the Wuxi CDC WOA which disseminates health information to the public. Users need to read and agree terms of use prior to log in. Gender, age, and regional distribution were indicated in individual profiles. There are three main approaches for WeChat users to read the articles. Users can obtain health information directly from WOAs as they subscribed as well as through “Moments”, a functional mode of WeChat by which users can see their friends’ posts. In addition, users can read the articles shared by friends. Two investigators who received training regarding the purpose of the study and the data collection procedures, used the same standard for classification of the variables throughout the study. We used double logging of data and conducted a consistency check for the collected data.

### Inclusion criteria

Non-COVID-19-related articles published by the Wuxi CDC WOA and user information dated from January 1, 2022 to December 31, 2022 (end of data collection) were included in this study.

### Variables and characteristics

All information was classified into different types of variables. The categorical variables include city, gender, age, type of articles, incentives, position of articles and the nature of articles. For those seeking enhanced privacy, WeChat provides tools to selectively reveal personal information while maintaining control over their online identity.According to the data exported from the WeChat official account, Users information were anonymous. City were divided into five categories by default(see addtional file 1): first tier city, second tier city, third tier city,fourth-tier city and under and unknown for those who revealed their personal information.Gender were divided into three categories:male, female and unknown.Age were divided into six categories by default: Less than 18,18–25, 26–35, 36–45, 46–60 and above 60. Based on the content of articles, we divided type of articles into five categories, including Infectious disease, Chronic disease, Environmental health, Nutrition and food-borne disease, and Health education and promotion. According to the copyright by the Wuxi CDC, the nature of articles were categorized into original and reproduced. The position of articles was categorized into headline and non-headline. Whether articles contained incentives(such as red envelopes or gifts) was categorized into yes and no.

Continuous variables included the number of images in article, the number of words in article, user engagement of followers 7 days after the article was published, previous studies used one or combination of “views”, shares, and “likes” or add to favorites as measurement of user engagement [16–19, 20, 21], researches indicated that sharing an article shows users’ interest in a certain health theme [22]. Liking a post reflects their preference and appreciation [19, 23]. After reading an article, users can like it to show their appreciation for the important message [24]. Still there are no unitive metrics of user engagement. In the end of 2019, the WeChat public account has launched a new function for operators only—100% reading completion rate,the number of people who read through to the end/total number of readings. Based on the WeChat official explanation [25], it can be used to directly measure the appeal of an article to readers. Since our numbers of “views” “likes” and add to favorites were as low as 20 or under per article, user engagement were measured by 100% reading completion rate and shares as our research dependent variables.

### Statistical analysis

Descriptive statistical analysis was performed for continuous variables. We performed multiple logistic regression, which was utilized in previous studies [26–29], to explore the influence factors on the user engagement. We transformed continuous dependent variables (shares and 100% reading completion rate) into binary variables, because the data did not follow a normal distribution. For the engagement of followers, the median volume was used as the cut-off point to distinguish“good engagement”(equal to or larger than the median) and“poor engagement”(less than the median).Poor engagement were set as the reference. For the nature of articles, reproduced articles were set as the reference. For incentives, articles not contain incentives were chosen as the reference. For the headline, non-headlines were set as the reference. For article type, health education and promotion articles were chosen as the reference. All independent variables: number of words, number of images, article type, incentives, article nature and position of article were included with Forward: Likelihood Ratio(LR) method. All analyses were performed using the statistical software package Microsoft IBM® SPSS® 20. Values are expressed as mean (SD), when an associated variable was a continuous variable or as the frequency and percentage when that was a discrete variable. Adjusted odds ratios (ORs) and corresponding 95% confidence intervals (CIs) for the factors were computed. *P* < 0.05 were defined as statistically significant.

## Results

As of the end of 2022, the Wuxi CDC WOA boasted a subscriber base of 891,170 individuals. Table 1 summarizes the demographic breakdown of this subscriber base, which reveals a female-majority audience (58.75%) with males comprising the remaining 39.3%. Notably, the majority of subscribers resided in third-tier cities (82.59%) and belonged to the 26–35 (43.63%) and 36–45 (30.6%) age groups.

**Table 1:** Characteristics of Wuxi CDC WOA subscribers

Variables	Count (*N* = 891,170)
City	
First tier	22,460(2.52%)
Second tier	61,236 (6.87%)
Third tier	735,999(82.59%)
Fourth tier and below	66,170 (7.42%)
unknown^1^	5314 (0.59%)
Gender	
male	349,856(39.3%)
female	523,576(58.75%)
unknown	17,738(1.99%)
Age	
Less than 18	5914(0.66%)
18–25	110,328(12.38%)
26–35	388,887(43.63%)
36–45	272,785(30.6%)
46–60	95,318(10.69%)
Above 60	18,118(2.03%)

A total of 170 articles were included in the analysis, of which 110 (64.7%) were original and 60 (35.3%) were reproduced. There were 32 (18.8%) Infectious disease articles, 20 (11.8%) Chronic disease articles, 23 (13.5%) Environmental health articles, 20(11.8%) Nutrition and Food-borne disease articles and 75(44.1%) Health education and promotion articles,respectively. The average number of images in articles was 9.86 (± 5.716), the average number of words in articles was 1086.4 (± 457.4), Additionally, 6.5% (*n* = 11) of the articles integrated incentive information or red envelopes as engagement strategies.

**Table 2:** Univariate logistic analysis of the effectiveness of health education dissemination (*n* = 170)

Variables	Mean ± SD/n (%)	100% reading completion rate		shares	
		OR (95% CI)	*P*	OR (95% CI)	*P*
Number of images	9.86 ± 5.716	0.884 (0.825,0.948)	< 0.001	0.977 (0.925,1.031)	0.389
Number of words	1086.4 ± 457.4	0.999 (0.998,1)	0.002	1.001 (1,1.001)	0.106
Article type			0.164		0.031
Infectious disease	32 (18.8)	0.482(0.208,1.119)	0.089	0.938 (0.408,2.518)	0.880
Chronic disease	20 (11.8)	0.470(0.172,1.284)	0.141	1.206 (0.449,3.237)	0.710
Nutrition and food-borne disease	23 (13.5)	1.321(0.499,3.497)	0.575	5.728 (1.778,18.458)	0.003
Environmental health	20 (11.8)	1.308(0.468,3.655)	0.608	0.649 (0.233,1.810)	0.409
Health education and promotion	75 (44.1)	1	ref	1	ref
Contain incentives			0.991		0.786
yes	11 (6.5)	0.991 (0.291,3.388)		1.185 (0.347,4.041)	
No	159 (93.5)	ref		ref	
Article nature			0.307		0.283
Original	110 (64.7)	1.395 (0.737,2.639)		1.414 (0.752,2.660)	
reproduced	60 (35.3)	ref		ref	
Postion of article			0.905		0.010
Headline	120 (70.6)	1.041(0.537,2.019)		2.454 (1.234,4.879)	
Non-Headline	50 (29.4)	ref		ref	

Univariate logistic regression analysis (Table 2) revealed a significant inverse association between 100% reading completion rate and image count (OR = 0.884, 95% CI = 0.825–0.948), word count (OR = 0.999, 95% CI = 0.998–1). This suggests that articles with a higher number of images or words were less likely to be read to completion. In addition, article placement in headlines (OR = 2.454, 95% CI = 1.234–4.879) and the topic of Nutrition and Foodborne diseases (OR = 5.728, 95% CI = 1.778–18.458) exhibited significant positive associations with article sharing behavior.

**Table 3:** Factors associated with 100% reading complete rate

Variables	β	S.E	Wals χ^2^	OR (95% CI)	*P* value
Number of images	-0.111	0.036	9.226	0.895(0.834,0.961)	0.0032
Number of words	-0.001	0.000	7.956	0.999 (0.998,1)	0.012

Table 3 shows the final logistic regression model, adjusting for all other included variables, identified the number of images and word count as significant predictors of the 100% reading completion rate (see Table 3). Specifically, each additional image in an article was associated with a 10.5% decrease in the odds of 100%complete reading (OR = 0.895, 95% CI: 0.832–0.962). Similarly, for every additional word, the odds of 100% complete reading decreased by 0.1% (OR = 0.999, 95% CI: 0.998-1.000). These findings suggest that articles with higher image or word count are less likely to be read in their entirety.

**Table 4:** Factors associated with shares

Variables	β	S.E	Wals χ^2^	OR (95% CI)	*P* value
**Article type**					0.018
Infectious disease	-0.451	0.457	0.976	0.637(0.260,1.558)	0.323
Chronic disease	-0.250	0.535	0.219	0.779(0.273,2.222)	0.640
Nutrition and food-borne disease	1.508	0.613	6.058	4.520(1.360,15.002)	0.014
Environmental health	-0.927	0.557	2.771	0.396(0.133,1.179)	0.096
health education and promotion	ref				
**Headlines**					
yes	1.083	0.400	7.327	2.952(1.348,6.466)	0.007
no	ref				

Table 4 reveals significant associations between article type, location, and sharing behavior. Subscribers were 4.52 times more likely to share articles related to nutrition and food-borne diseases compared to regular health education and promotion articles. Additionally, articles featured in Headlines were shared 1.95 times more often than non-highlighted articles.

## Discussion

A substantial body of research supports the efficacy of the Health Belief Model (HBM) as a framework for healthcare interventions. Studies across diverse contexts have demonstrated its effectiveness in promoting behavior change related to risk assessment, disease transmission mitigation, and self-health management [30–32]. With the guidance of HBM model, the health promotion of our WOA lies in its ability to address individual perceptions and beliefs regarding susceptibility, severity, and potential benefits of preventative or management actions. By tailoring information and interventions to these specific factors, we can empower individuals to make informed decisions and adopt healthy behaviors, ultimately leading to improved individual and population health outcomes.

While increasing the opening rate of WeChat public accounts remains crucial for broadening reach and disseminating health knowledge to new audiences, a more profound challenge lies in effectively retaining existing subscribers and maximizing the impact of health education for them. Maintaining consistent engagement, tailoring content to diverse needs, and overcoming information overload require a deeper understanding of subscriber behavior and preferences.

In today’s reading environment, where young people are increasingly seeking “short, concise, and quick” reading, it is imperative to explore strategies for consistently producing engaging works that captivate readers’ attention, all the while upholding a distinct brand and fostering innovation [33]. Therefore, fostering user engagement is crucial for the effective dissemination of information and health promotion; the identification of predictors of social media engagement can inform the develop [34]. User engagement is widely recognized as a critical determinant of effectiveness for behavior change. Research consistently demonstrates a positive association between higher engagement levels and improved health outcomes [35–37]. However, simply maximizing engagement may not be the most valuable approach. Instead, promoting “effective engagement” might be more beneficial [38]. This concept implies an empirically defined level of engagement necessary to achieve the intervention’s intended outcomes. By prioritizing the quality and intensity of engagement rather than sheer quantity, interventions can be better targeted towards individuals most likely to experience positive behavior change.

Currently, there is a lack of evaluation indicators for the quality and audience acceptance of health communication articles in China, making it difficult to scientifically measure the effectiveness of communication [39]. Evaluating the effectiveness of health education dissemination on WeChat public accounts has been hampered by the lack of unified, reliable metrics for user engagement. While past studies have relied on combinations of views, shares, and likes, these metrics often capture superficial engagement or are susceptible to manipulation. This study addresses this gap by proposing 100% reading completion rate as a new, potentially more meaningful indicator of engagement. This metric goes beyond mere exposure to assess the depth of user engagement with the content, offering valuable insights into message delivery effectiveness. While acknowledging potential limitations associated with skimming or partial reading, 100% completion rate provides a clearer picture of how users interact with and internalize health information compared to existing metrics. Additionally, previous studies largely neglected investigating this metric due to limitations in data access. By utilizing novel scraping techniques and collaborating with the Wuxi CDC, this study overcomes these restrictions and opens new avenues for evaluating health education dissemination through WeChat public accounts.

This study revealed that while article length and image count influenced 100% reading completion rate, which have shown that people in the new media era are more likely to use fragmented time to obtain information and perspectives. The greatest characteristic of fragmented reading is that it is “shallow " [40]. However, we should approach their optimization cautiously. Zhang et al. found that articles containing 1,000 to 1,500 words and 1,500 to 2,000 words were more likely to obtain high-level reading and liking [41]. That might due to the differences of our subscriber characteristics and reading habits. In addition, there were barely researches reporting images as an influence factor for content analysis. Only Yin et al. found a combination of text, links and pictures was associated with a higher rate of reading compared to text only articles [14]. The inverse association between image account and good user engagement might due to poor image qualities such as out-fashioned pictures, images size issues or irrelevant to the content.Thus, further content analysis are need to determine the best words and images counts as well as qualities. Simply shortening articles or limiting images could sacrifice information depth or visual engagement. Instead, future improvements should consider strategies like effective content structuring, strategic image integration, and offering diverse formats like summaries or videos. Moreover, optimal content length and visuals likely vary depending on the target audience and information type. Additionally, the publication of articles in public accounts should reasonably arrange the publication time and article proportion, which is in line with people’s habit of reading anytime and anywhere with fragmented time. Information services need to focus on attention resources to achieve long-term development [39, 42]. This study’s finding that article type was not associated with 100% reading completion rate suggests that while overarching themes may not directly influence users to finish reading. This underscores the need for in-depth content analysis in future research, focusing on identifying engaging writing styles, impactful information presentation formats, and audience-tailored language. By understanding what aspects of content truly “attract users’ appetite,” we can inform the creation of targeted, engaging health education materials that resonate with diverse audiences and ultimately maximize the effectiveness of health promotion dissemination.

Investigations have found that over 70% of Chinese people obtain health education through the internet, and nearly 30% of them choose to search for health information through WeChat searches. At the same time, nearly one-third of people often read health information through WeChat Moments, public accounts, or chatting groups [43]. WeChat can be used to target information to specific audiences and to spread information through users’ relatively stable social relationships [40]. Passive exposure through WeChat Moments and chatting groups has become an important channel for many WeChat users to obtain health information [44]. This also verifies that information sharing based on close social relationships can effectively improve the breadth and depth of communication. Zhu et al. [45] showed that if the users of WeChat public accounts can give sufficient recognition to the content of the article, they will usually share the article with people around them or read it multiple times by forwarding the article. Previous studies showed that Diffusion of innovation theory posits that the dissemination of novel ideas, such as vaccine adoption, is amplified by interpersonal influence.peers are required to induce individual acceptance [46–48]. “shares “ in WOA, allows potential opinion leader to disseminate their ideas.Our study identified significant differences in article sharing behavior among subscribers. Articles related to Nutrition and food-borne diseases were shared 352% more compared to regular Health education and promotion articles, suggesting audiences prioritize content perceived as personally relevant or potentially impactful. Similarly, articles featured in Headlines were shared 195.2% more often, highlighting the effectiveness of strategic placement in attracting attention and encouraging dissemination. The result collaborated with Ji H. findings: the content of articles was correlated to the users’ engagement and was identified as an essential factor to determine whether WeChat users forward or share articles with friends [29]. Card KG’s researches also found that other social media platforms have also shown that the content of posts appears to have a significant effect on user engagement [49]. These findings suggest that tailoring content to address personal concerns and utilizing prominent platform features can be powerful strategies for maximizing the reach and impact of health education messages on WeChat, which would ultimately impact broader public health outcomes.

Our analysis revealed surprisingly low engagement with infectious disease and chronic disease articles, as evidenced by their significantly lower completion rates and sharing compared to other themes. This phenomenon can be explained by the research of MEFFERT et al. [44, 50], who found that authoritative information related to policies, data updates, and scientific knowledge is the least popular on social media. This may be because authoritative information is often long, requires a certain level of knowledge to understand and is not humorous [51].

How social media can best be used to achieve health information dissemination and public health outcomes is a topic of much discussion and study in the public health community. Acquisition and dissemination of health information play a significant role in promoting positive health behavior change [52]. In the era of content is king, health-related WeChat public accounts need to dynamically assess the health education needs of their target audiences and provide specific and targeted health information and guidance. This is also the core element for improving the willingness of professional people to use WeChat public accounts and then increasing their usage frequency [53]. Therefore, it is crucial to dynamically monitor and assess the needs of WeChat public account platform audiences and adjust the content and operating strategies of articles in a timely manner in future work. Understanding social media activities can provide new ways for public health decision-makers, practitioners, and researchers to identify social and behavioral barriers to infection control and promote communications between public health agencies and the public. This will ultimately help to determine the best intervention strategies [54, 55].

## Limitations

This study collected information from WeChat official accounts against the background of the COVID-19 pandemic. Although COVID-19-related information has been excluded, the reading habits of the audience may change due to changes in daily life, work, social environment, and the focus of health education in the post-pandemic era. Therefore, further studies are needed to validate the gold standard for dissemination effects.

This study is limited to Wuxi CDC WOA subscribers, which may affect the generalizability of the conclusions to other health education-related WeChat public platforms in other provinces and countries. More research is needed to build a model that predicts the optimal number of images and words and other aspects that can maximize health education dissemination.

### Electronic supplementary material

Below is the link to the electronic supplementary material.

**Additional file 1: Table S1.** Variable Description: A table shows the explanation of variables we selected in this study

## Data Availability

The data that support the findings of this study are available from Wuxi CDC, but restrictions apply to the availability of these data, which were used under license for the current study and thus are not publicly available. Data are, however, available from the authors upon reasonable request and with the permission of the Wuxi CDC.

## Footnotes

1 For those seeking enhanced privacy, WeChat provides tools to selectively reveal personal information while maintaining control over their online identity.
