# Publisher Correction: Effects of Wuxi CDC WeChat official account article features on user engagement in health promotion

**DOI:** 10.1186/s12889-024-18434-9

**Published:** 2024-04-08

**Authors:** Xinyi Yin, Junxia Pan, Fanfan Xu

**Affiliations:** Department of Health Promotion, Wuxi Center for Disease Control and Prevention, The Affiliated Wuxi Center for Disease Control and Prevention of Nanjing Medical University, 499 Jincheng Road, 214023 Wuxi, China

**Correction: BMC Public Health (2024) 24:756**

10.1186/s12889-024-18277-4

In the original publication of this article the author name Junxia Pan was incorrectly duplicated. The article has been updated to correct this error.

