# Supplementary material for: Effects of Wuxi CDC WeChat official account article features on user engagement in health promotion
Source: BMC Public Health. 2024 Mar 11;24:756. doi: 10.1186/s12889-024-18277-4 (PMC10929147; doi:10.1186/s12889-024-18277-4)
Supplement: Supplementary file 1 — Additional file 1: Table S1. Variable Description: A table shows the explanation of variables we selected in this study [file 12889_2024_18277_MOESM1_ESM.docx]

Title: Effects of Wuxi CDC WeChat Official account article features on user engagement in health promotion

Authors: Xinyi Yin, Junxia Pan, Fanfan Xu

**Table S1. Variable Description**

| **Item** | **Definition** |
| --- | --- |
| **I.Location of articles** |  |
| Headlines | The first of each push are headlines |
| Non-Headlines | Behind the main push among consecutive posted articles |
| **Ⅱ.Article content** |  |
| Infectious disease | Topic is related to infectious diseases except COVID-19. |
| Chronic diseases | Topic is related to chronic diseases, such as  diabetes, cancer, COPD,etc. |
| Food safety and nutrition | Topic is related to food safety or nutrition. For  example, sugar and salt intake, BMI. |
| Environmental and occupational health | Topic is related to air hygiene, drinking water  hygiene, soil hygiene, housing hygiene, occupational diseases, etc. |
| Health education and promotion | Topic is related to events organized to conduct  health education(World No Tobacco Day, World AIDS Day,etc) and popular knowledge of life, such as how to manage weight, the benefits of quit smoking, etc. |
| **Ⅲ.City** |  |
| First-tier city | Metropolises that occupy an important position in national political, economic and other social activities and have a leading role and radiation driving ability. |
| Second-tier city | Most of the second-tier cities are provincial capitals, open coastal cities and economically developed prefecture-level cities in the central and eastern regions |
| Third-tier city | Small and medium-sized cities that are strategically important or relatively developed or have a large economic aggregate |
| Fourth-tier city | Mainly prefecture-level cities in the central region, but also include county-level cities in economically developed areas of some eastern provinces and prefecture-level cities and county-level cities in the west |

*Vaccination and organizational activities were not listed since we settled individual modules for service purpose.
